# Interactions between the gut micro-community and transcriptome of *Culex pipiens pallens* under low-temperature stress

**DOI:** 10.1186/s13071-022-05643-7

**Published:** 2023-01-12

**Authors:** Wen-Xiang Lv, Peng Cheng, Jing-Jing Lei, Hui Peng, Chuan-Hui Zang, Zi-Wei Lou, Hong-Mei Liu, Xiu-Xia Guo, Hai-Yang Wang, Hai-Fang Wang, Chong-Xing Zhang, Li-Juan Liu, Mao-Qing Gong

**Affiliations:** Department of Medical Entomology, Shandong Institute of Parasitic Diseases, Shandong First Medical University & Shandong Academy of Medical Sciences, Jining, 272033 Shandong People’s Republic of China

**Keywords:** 16S rRNA, Transcriptome, *Culex pipiens pallens*, Low temperature, Interaction

## Abstract

**Background:**

*Culex pipiens pallens* (Diptera: Culicidae) can survive at low temperature for long periods. Understanding the effects of low-temperature stress on the gut microflora and gene expression levels in *Cx. pipiens pallens*, as well as their correlation, will contribute to the study of the overwintering mechanism of *Cx. pipiens pallens*.

**Methods:**

The gut bacteria were removed by antibiotic treatment, and the survival of *Cx. pipiens pallens* under low-temperature stress was observed and compared with the control group. Then, full-length 16S rRNA sequencing and the Illumina HiSeq X Ten sequencing platform were used to evaluate the gut microflora and gene expression levels in *Cx. pipiens pallens* under low-temperature stress.

**Results:**

Under the low-temperature stress of 7 °C, the median survival time of *Cx. pipiens pallens* in the antibiotic treatment group was significantly shortened by approximately 70% compared to that in the control group. The species diversity index (Shannon, Simpson, Ace, Chao1) of *Cx. pipiens pallens* decreased under low-temperature stress (7 °C). Non-metric multidimensional scaling (NMDS) analysis divided all the gut samples into two groups: control group and treatment group. *Pseudomonas* was the dominant taxon identified in the control group, followed by *Elizabethkingia* and *Dyadobacter*; in the treatment group, *Pseudomonas* was the dominant taxon, followed by *Aeromonas* and *Comamonas*. Of the 2417 differentially expressed genes (DEGs), 1316 were upregulated, and 1101 were downregulated. Functional GO terms were enriched in 23 biological processes, 20 cellular components and 21 molecular functions. KEGG annotation results showed that most of these genes were related to energy metabolism-related pathways. The results of Pearson’s correlation analysis showed a significant correlation between the gut microcommunity at the genus level and several DEGs.

**Conclusions:**

These results suggest that the mechanism of adaptation of *Cx. pipiens pallens* to low-temperature stress may be the result of interactions between the gut bacterial community and transcriptome.

**Graphical Abstract:**

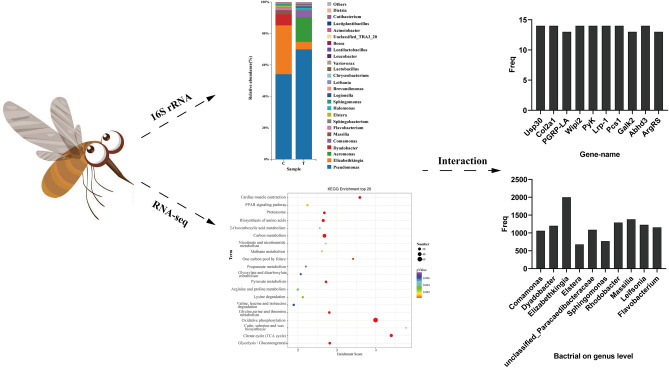

**Supplementary Information:**

The online version contains supplementary material available at 10.1186/s13071-022-05643-7.

## Background

*Culex pipiens pallens* belongs to the order Diptera and family Culicidae. It is mainly distributed at a latitude of 33°N in China [1] and is the main transmission vector of Japanese encephalitis virus and *Bancroftian filariasis* [2, 3]. *Culex pipiens pallens* mainly overwinter as adults, and the overwintering mosquitoes play an important role in the spread and prevalence of mosquito-borne diseases [4]. Therefore, it is crucial to clarify the cold tolerance mechanism of *Cx. pipiens pallens* to improve mosquito control strategies.

Temperature is one of the important factors affecting the geographical distribution and diffusion of insects [5]. Low temperature limits the metabolism of insects and even causes cell dehydration and tissue damage in serious cases, posing great challenges to insect survival [6, 7]. In the cold winter months, insects undergo a series of physiological and biochemical reactions to improve their cold tolerance to adapt to changes in the low-temperature environment. Previous studies have shown that the overwintering mechanism of insects mainly includes reducing the water content in their bodies to increase body fluid concentrations and reduce the freezing point of body fluids and improving cold resistance by accumulating small molecular substances such as fat and glycogen [8]. In addition, antifreeze proteins (AFPs), heat shock proteins (HSPs) and other proteins also play an important role in the regulation of insect cold resistance [9], and the mechanism of adaptation to low temperature remains the focus of insect research. In recent years, the low-temperature adaptation mechanism of insects has been studied mainly at the transcriptome level [10–12], and it has been found that some genes related to immunity and stress response play an important role in the cold resistance of insects. Recent studies have found that the gut microbiota of insects changes with temperature and plays a role in host resistance to cold stress. The intestinal symbiotic bacterium *Klebsiella michiganensis* BD177 enhances the resistance of *Bactrocera dorsalis* to cold stress by stimulating the host arginine and proline metabolic pathways and affecting mitochondrial function [13]. Coincidentally, *Drosophila melanogaster* grown at low temperature (13 °C) has the strongest cold tolerance and the highest abundance of *Wolbachia*, while *D. melanogaster* grown at high temperature (31 °C) has the strongest heat tolerance and the highest abundance of *Acetobacter* [14].

Gut bacteria play an important role in insect growth and development, digestion and absorption, nutrient metabolism and immune defense [15–18]. In addition, recent studies have shown that changes in the gut microbiota can affect the expression of many genes in the gut and body tissues [19, 20], and vice versa [21]. The gut symbiotic bacterium *Burkholderia* stimulates insect growth and egg-laying by regulating the expression of insect storage proteins and vitellogenin genes [19]. *Drosophila* modulates the richness and diversity of the gut microbiota by regulating the expression of genes encoding two immune effectors, antimicrobial peptides and lysozyme [20]. Compared with aseptically reared willow leaf beetle larvae, the immunity-related genes encoding peptidoglycan recognition protein (PGRP), defensins and prophenoloxidase (PPO) are all upregulated in the body tissue and intestinal tract of conventionally reared willow leaf beetle larvae, suggesting that local defense and systemic immunity play important roles in maintaining intestinal homeostasis [21].

There have been extensive studies on the gut bacteria of mosquitoes, mainly regarding the effect of gut bacteria on mosquito development and diseases [22–24]. The role of gut bacteria in mosquito resistance to low temperature has not been reported. In this study, 16S rRNA sequencing and transcriptome sequencing were used to analyze the effects of low-temperature stress on the intestinal microbiota and gene expression in *Cx. pipiens pallens*, as well as their correlation, and to explore the mechanism underlying adaptation of *Cx. pipiens pallens* to low-temperature stress to provide a reference for the overwintering mechanism of *Cx. pipiens pallens*.

## Methods

### Rearing of mosquitoes

*Culex pipiens pallens* were provided by Shandong Institute of Parasitic Diseases; this strain has been maintained in the mosquito breeding room of Shandong Institute of Parasitic Diseases for more than 20 years. The feeding conditions were as follows: temperature of 26 ± 2 °C; relative humidity of 75 ± 5% and an L:D photoperiod = 14:10 h. Larvae were fed pig liver powder and yeast powder (1:3), and adult mosquitoes were fed a 10% glucose solution.

### Antibiotic treatment

After initial antibiotic sensitivity tests, a 10% sucrose solution containing a mixture of streptomycin (50 μg/ml) and penicillin (50 μg/ml) was administered from the first day of emergence for 5 days, after which the antibiotic solution was replaced with sterile water [25]. To determine the efficacy of antibiotic treatment, 10 *Cx. pipiens pallens* were randomly selected to prepare an intestinal suspension and inoculated on Luria-Bertani (LB) agar plates, which were then incubated at 30 °C for 48 h to observe colony growth in the antibiotic treatment group. The treatment effect was verified by performing Accu16S™ absolute bacterial quantification (Haotian Biotech Co., Ltd.). *Culex pipiens pallens* reared at conventional temperature (26 °C) was established as the control group, and the effects of antibiotic treatment on the normal life activities of *Cx. pipiens pallens* were observed.

### Survival analysis of conventionally reared and antibiotic-treated *Cx. pipiens pallens* at low temperature

The antibiotic-treated group and the conventionally reared group were placed in 20 cm × 20 cm × 20 cm cages (there were 70 female *Cx. pipiens pallens* in each cage and 3 biological replicates in each group) and provided with 10% sugar water. Antibiotic-treated and conventionally reared *Cx. pipiens pallens* were placed at 4 °C, 7 °C and 10 °C to investigate the role of the gut microbiome in host resistance to temperature stress. The death of *Cx. pipiens pallens* was recorded every 24 h until all mosquitoes died to determine the lowest temperature at which the median survival time decreased significantly.

### Gut sampling

The median survival time of the antibiotic treatment group at 7 °C was 6 days, and thus we chose 6 days as the main time point for subsequent studies, including 16S rRNA sequencing and transcriptome sequencing. Female mosquitoes exposed to a low temperature (7 °C) and normal temperature (26 °C) were randomly soaked in 75% ethanol for 3 min and washed with sterile distilled water for 2 min. After dissection under aseptic conditions, the midgut was removed and placed into a 1.5-ml aseptic collection tube. Twenty mid-gut samples were collected as a group, with four biological replicates in each group. The samples were then immediately frozen in liquid nitrogen and subsequently stored at − 80 °C until further analysis.

### DNA extraction, full-length 16S rRNA sequencing and community analysis

Total DNA was extracted from gut tissues with the NucleoSpin^®^ 96 Soil Kit (Macherey–Nagel, Germany) according to the manufacturer’s instructions. The primer sequences of the full-length 16S rRNA were as follows: 27F (AGRGTTTGATYNTGGCTCAG) and 1492R (TASGGHTACCTTGTTASGACTT). The PCR system (10 μl) contained the following: forward primer, 0.3 μl; reverse primer, 0.3 μl; KOD FX Neo Buffer, 5 μl; dNTPs (2 mM each), 2 μl; KOD FX Neo, 0.2 μl; DNA, 50 ng; ddH_2_O added to achieve a final volume of 10 μl. PCR was performed as follows: 95 °C for 5 min; 25 cycles of 95 °C for 30 s, 50 °C for 30 s, and 72 °C for 40 s; and 72 °C for 7 min. The PCR products were purified, quantified, and homogenized to form a sequencing library (SMRTbell). The established library was inspected first, and the qualified library was sequenced by a PacBio Sequel system [26]. PacBio Sequel generates circular consensus sequencing (CCS) files in bam format using SMRTlink analysis software. Data for different samples were identified according to the barcode sequence and converted to fastq format. CCS reads were identified based on barcode sequences by Lima v1.7.0, generating raw CCS sequences. Primer sequences were identified and removed by Cutadapt 1.9.1 [27]. Raw-CCS sequences were filtered based on length, which generated Clean-CCS sequences. Chimeric sequences were identified and removed using UCHIME v4.2 [28], generating effective CCS sequences.

USEARCH software [29] was used to cluster reads at a similarity level of 97.0% and obtain the operational taxonomic units (OTUs). OTU classification annotations were based on the Silva (http://www.arb-silva.de) database. QIIME [30] was applied to determine the abundance of each species in the samples, and a distribution histogram at each taxonomic level and Venn diagrams were generated using an R package. The abundance and diversity of the microbial communities were analyzed by the alpha diversity index. The non-metric multidimensional scaling (NMDS) analysis was based on the OTU abundance of sequenced samples, and Bray-Curtis dissimilarities were calculated using the “vegan” package in R.

### RNA isolation and Illumina sequencing

Four groups of female *Cx. pipiens pallens* reared at normal temperature (control groups) and four groups reared at a low temperature treated at 7 °C (experimental groups) were selected for the transcriptome analysis (A was the control group and B was the experimental group, with 20 female mosquitoes in each group). Total RNA was extracted using the QIAGEN RNeasy Mini Kit (Qiagen, Germany) according to the manufacturer’s protocol. RNA purity and quantity were evaluated using a NanoDrop 2000 spectrophotometer (Thermo Scientific, USA). RNA integrity was assessed using an Agilent 2100 Bioanalyzer (Agilent Technologies, Santa Clara, CA, USA). Then, libraries were constructed using the TruSeq Stranded mRNA LT Sample Prep Kit (Illumina, San Diego, CA, USA) according to the manufacturer’s instructions. Transcriptome sequencing and analysis were conducted by OE Biotech Co., Ltd. (Shanghai, China).

The libraries were sequenced on an Illumina HiSeq X Ten platform, and 150-bp paired-end reads were generated. Raw data (raw reads) in fastq format were first processed using Trimmomatic [31], and the low-quality reads were removed to obtain clean reads. HISAT2 [32] was then used to compare clean reads with the specified reference genome (*Cx. pipiens quinquefasciatus* genome) to determine the location on the reference genome or gene and the sequence characteristics of the sequenced samples.

### Differential expression analysis and GO/KEGG enrichment analysis

The reads of each sample mapped to reference genes were assembled using StringTie v1.3.3b. Then, the fragments per kilobase of the transcript sequence per millions mapped reads (FPKM) of each gene were calculated based on the length of the gene and read counts mapped to this gene. The FPKM [33] value of each gene was calculated using Cufflinks [34], and the read counts of each gene were obtained using HTSeqcount [35]. Differential expression analysis was performed using the DESeq (2012) R package. A *P* value < 0.05 and fold change > 2 or fold change < 0.5 were set as the thresholds for significant differential expression. Hierarchical cluster analysis of differentially expressed genes (DEGs) was performed to determine the expression patterns of genes in different groups and samples. GO enrichment and KEGG [36] pathway enrichment analyses of DEGs were performed using R based on the hypergeometric distribution.

### Statistical and bioinformatics analysis

The survival rate of conventionally reared and antibiotic-treated *Cx. pipiens pallens* at low temperature was analyzed using the log-rank (Mental-Cox) test. The abundance of the gut bacterial community of *Cx. pipiens pallens* at ambient temperature and low temperature was analyzed using the t-test. Pearson correlation analysis was used to infer the correlation between the genus-level microbial community and host gene expression. All analyses were performed using GraphPad Prism or SPSS 22 statistical software. The results were shown as the means ± standard errors (SE), and *P* < 0.05 was considered statistically significant.

## Results

### Effects of antibiotics on *Cx. pipiens pallens*

**Fig. 1:**
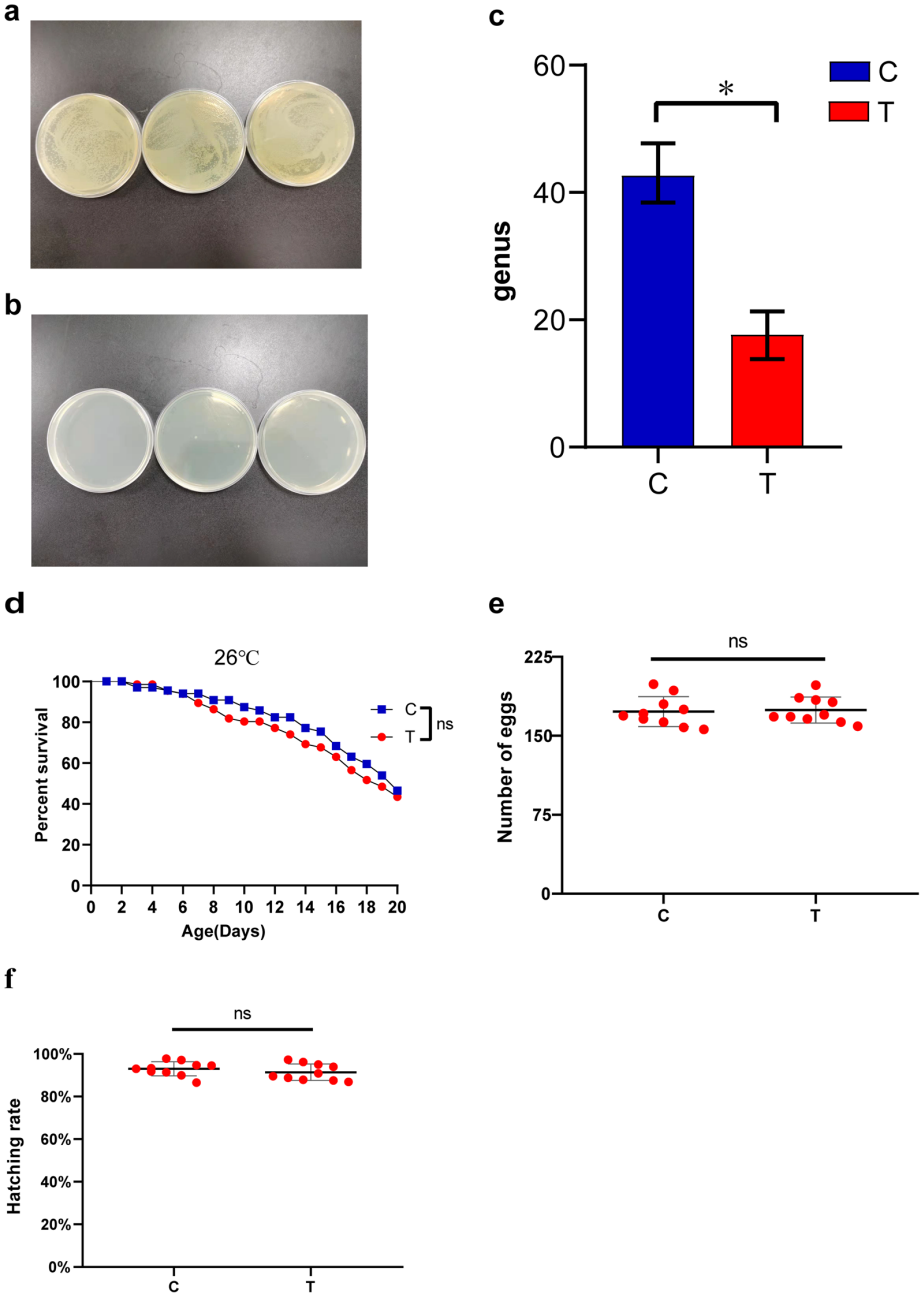
Diagram of the effects of antibiotic treatment. **a** Bacterial colony growth from the gut homogenate of the control group on an LB agar plate. **b** Bacterial colony growth from the gut homogenate of the antibiotic treatment group on an LB agar plate. **c** Absolute quantitation of bacterial diversity by Accu16S™ at the genus level. **d** Antibiotic treatment did not affect the survival rate of *Cx. pipiens pallens*. **e** Number of eggs laid. **f** Hatching rate. Three biological replicates were examined, and significant differences were determined using Student’s t-test and log-rank test; *P < 0.05. The letters C and T represent the control and antibiotic-treated groups, respectively

Antibiotic-treated intestinal homogenates were coated on LB agar plates for incubation at 30 °C for 48 h. The effect of removal of intestinal microorganisms is shown in Fig. 1a and b, and the results were verified by Accu16S™ absolute bacterial quantification (genus level, t-test, *t*_(2.093)_ = 5.653, *P* = 0.027) (Fig. 1c). Survival assays showed that antibiotic treatment had no effect on mosquito survival (log-rank test, *χ*^2^ = 0.3921, *df* = 1, *P* = 0.636) (Fig. 1d). The number of eggs (t-test, *t*_(18)_ = 0.235, *P* = 0.817) and hatchability (t-test, *t*_(18)_ = 0.994, *P* = 0.333) of mosquitoes treated with antibiotics were not significantly different from those of control mosquitoes (Fig. 1e and f). These results indicated that antibiotic treatment did not affect the normal life activities of mosquitoes, and thus they were subjected to further experiments.

### Survival analysis of conventionally reared and antibiotic-treated *Cx. pipiens pallens* at low temperature

**Fig. 2:**
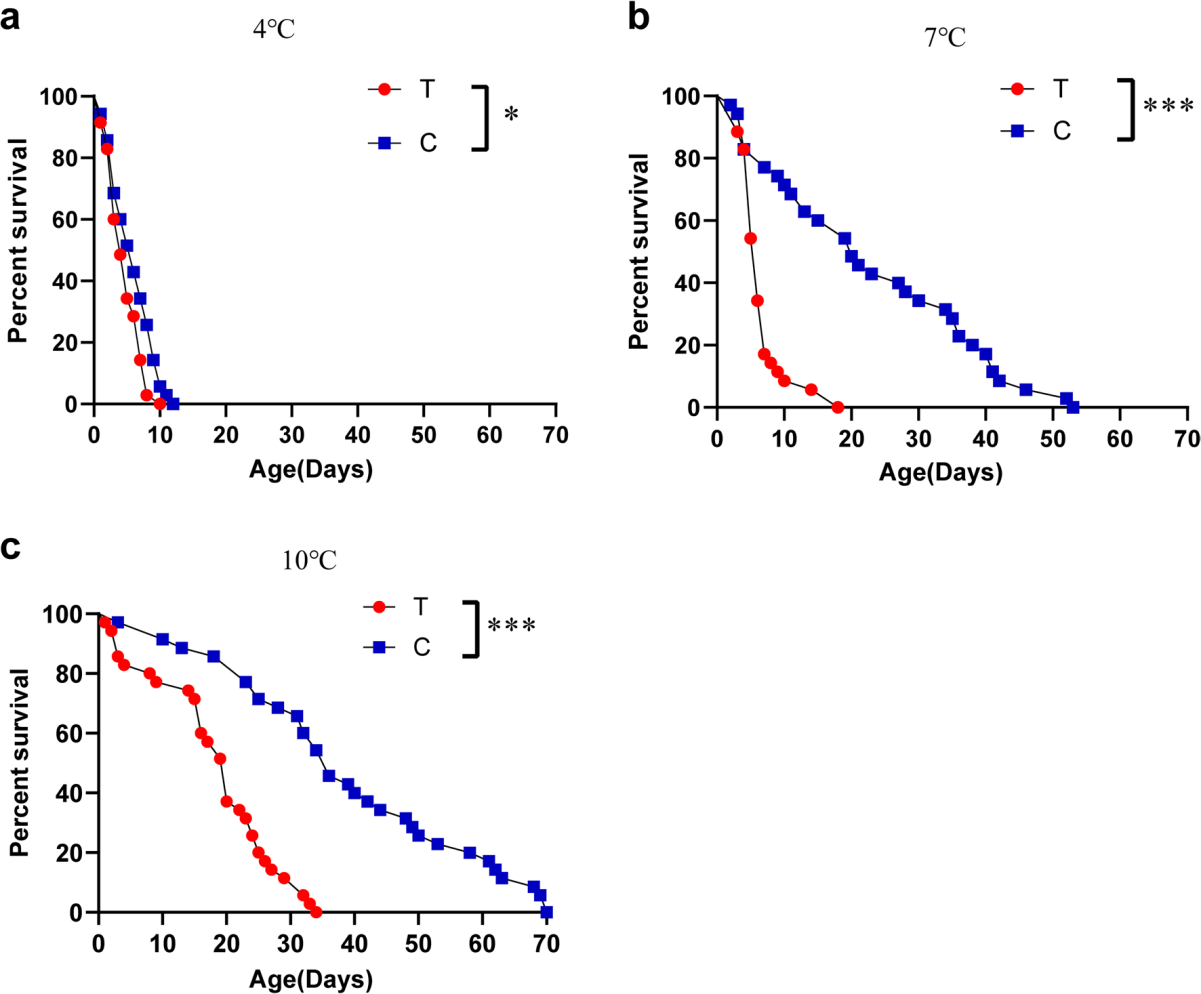
Survival curves of conventionally reared and antibiotic-treated *Cx. pipiens pallens* at low temperatures. **a** Survival curve of *Cx. pipiens pallens* at 4 °C. **b** Survival curve of *Cx. pipiens pallens* at 7 °C. **c** Survival curve of *Cx. pipiens pallens* at 10 °C. Significant differences were determined using the log-rank test: *P < 0.05, **P < 0.01, and ***P < 0.001. The letters C and T represent the control and antibiotic-treated groups, respectively

The survival of *Cx. pipiens pallens* at various temperatures is shown in (Fig. 2a–c). Statistically significant differences in the survival curves were observed between the antibiotic treatment group and the control group at 4 °C (log-rank test, *χ*^2^ = 9.389, *df* = 1, *P* = 0.0022), 7 °C (log-rank test, *χ*^2^ = 34, *df* = 1, *P* < 0.0001) and 10 °C (log-rank test, *χ*^2^ = 34.61, *df* = 1, *P* < 0.0001); 7 °C was the lowest temperature at which the median survival time of *Cx. pipiens pallens* was significantly different between antibiotic treatment and conventional treatment. The median survival time of *Cx. pipiens pallens* was significantly reduced from 20 days in the control group to 6 days, which was an approximately 70% reduction.

### Gut bacterial analysis of *Cx. pipiens pallens* under low-temperature stress

**Fig. 3:**
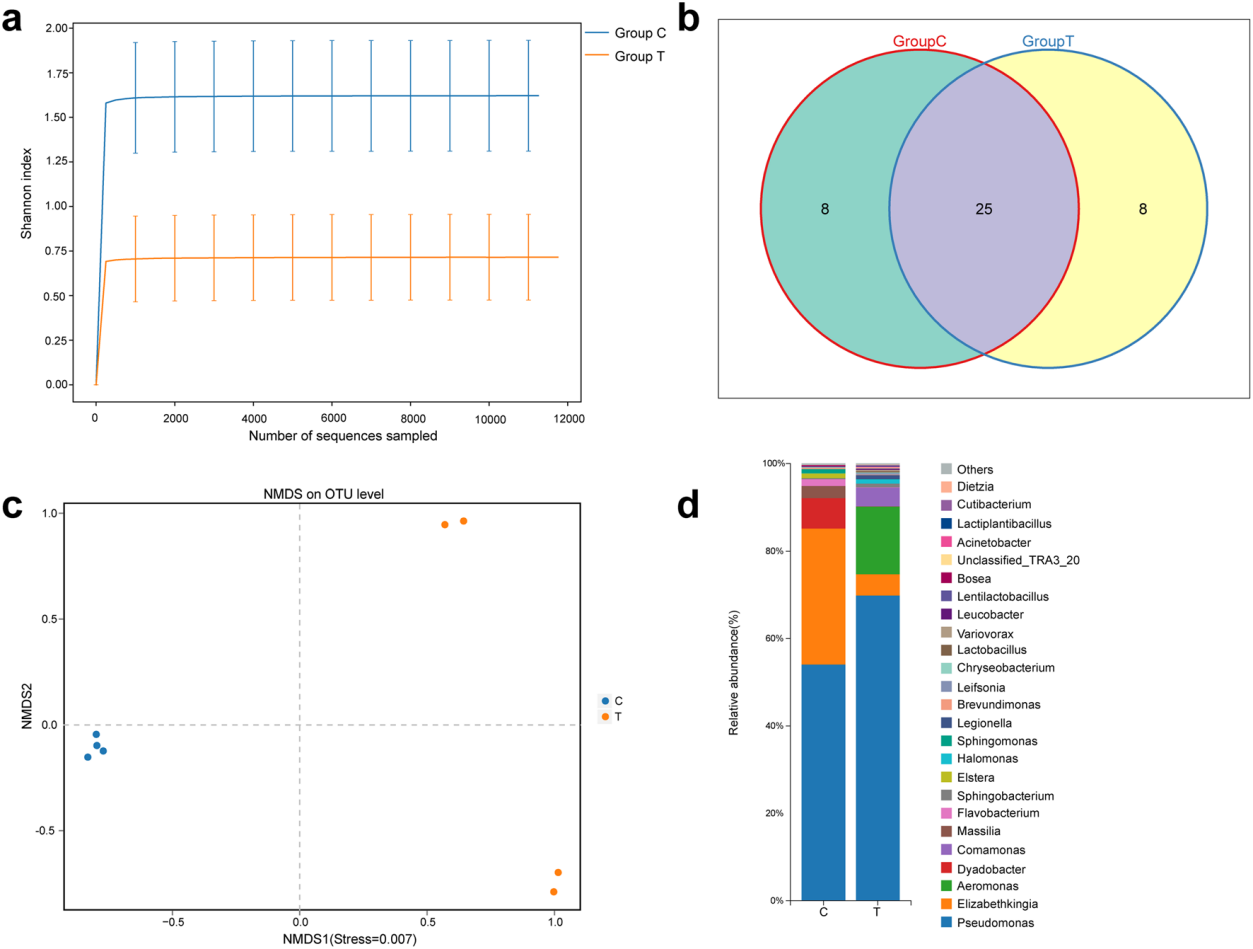
Analysis of the gut microbiota of *Cx. pipiens pallens* under low-temperature stress. **a** Dilution curve of the Shannon diversity index of the gut bacterial community in the control and treatment groups. **b** Venn diagram of the intestinal bacterial community in the control and treatment groups. **c** NMDS analysis of the control and treatment groups. **d** Histogram comparing relative bacterial abundance at the genus level between the control and treatment groups. Significant differences were determined using Student’s t-test. The letter C represents the control sample, and the letter T represents the low-temperature treated sample

The PacBio sequencing platform was used for paired-end sequencing of the amplicons of the V1–V9 region of the 16S rRNA gene. Sequencing of eight samples yielded a total of 103,385 CCS sequences through barcode identification. At least 12,382 CCS sequences were generated for each sample, with an average of 12,923 CCS sequences. Based on 97% sequence similarity, a homology alignment was performed on all sequences to identify OTUs, and the results were annotated to obtain 4 phyla, 5 classes, 18 orders, 28 families, 36 genera and 41 species (Additional file 1: Table S1). The Shannon diversity index dilution curve showed that with the increase in the number of sampled sequences, the number of species observed in each group of samples tended to be flat and saturated, indicating that the sequencing data were sufficiently complete and could be used for subsequent analysis (Fig. 3a). Twenty-five shared bacterial taxa were identified in the treated and control groups (Fig. 3b). The authenticity of the sequencing data was evaluated by calculating the sample coverage, and the results showed that the coverage was 0.98, indicating that the sequencing data were credible. The Chao1 and Ace indices were used to measure species abundance, namely, the number of species, and the Shannon and Simpson indices were used to measure species diversity. The results showed lower species abundance and diversity of *Cx. pipiens pallens* exposed to low temperature than those of the control group (Table 1).

**Table 1:** Alpha_diversity

Sample	Shannon	Simpson	Ace	Chao1	Coverage
C1	1.69	0.61	23.88	22.00	0.99
C2	2.24	0.70	26.58	26.00	0.99
C3	1.51	0.53	24.87	24.00	0.99
C4	1.77	0.61	36.05	28.75	0.99
T1	0.05	0.01	23.08	16.00	0.99
T2	1.95	0.54	29.41	45.00	0.99
T3	1.38	0.42	22.71	21.75	0.99
T4	1.55	0.47	24.31	16.33	0.98
	1.80	0.61	27.85	25.19	0.99
	1.23	0.36	24.87	24.77	0.99

The letters C and T represent the control and antibiotic-treated groups, respectively

Based on the OTU annotation, NMDS analysis was performed to divide all samples into two groups: treatment group and control group (PERMANOVA, *R*^2^ = 0.456, *df* = 1, *P* = 0.026) (Fig. 3c). At the genus level, *Pseudomonas* was dominant in the control group, followed by *Elizabethkingia* and *Dyadobacter*; *Pseudomonas* was also dominant in the treatment group, followed by *Aeromonas* and *Comamonas*. The abundance of *Elizabethkingia* in the treatment group was significantly lower than that in the control group (t-test, *t*_(6)_ = 7.697, *P* < 0.001) (Fig. 3d)**.**

### Transcriptome analysis of *Cx. pipiens pallens* under low-temperature stress

We performed reference transcriptome sequencing on eight samples and obtained a total of 56,112,953,542 clean data points. The number of effective reads for each sample ranged from 6,519,300,555–7,391,877,750, the Q30 base distribution was 92.2–92.7%, and the average GC content was 51.7%. By aligning reads to the reference genome, the genome alignment of each sample was obtained, and the alignment rate was 71.9–79.7% (Additional file 1: Table S2)**.**

**Fig. 4:**
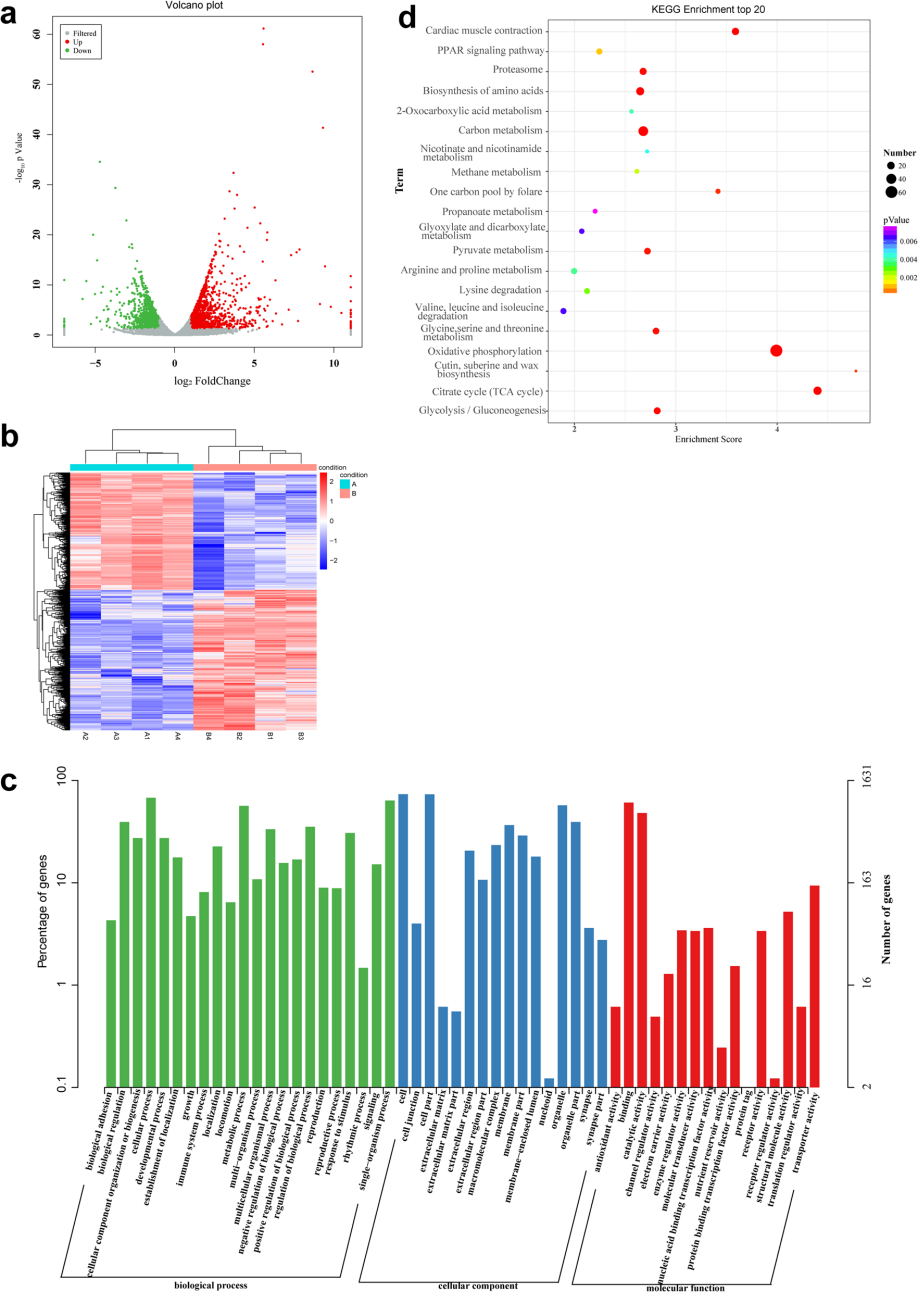
Analysis of DEGs. **a** DEGs identified in *Cx. pipiens pallens* treated at low temperature compared with those of the control group. Red dots represent upregulated genes, and green dots represent downregulated genes. **b** Cluster diagram of the DEGs between *Cx. pipiens pallens* exposed to low temperature and the control group. **c** Comparison of the distribution of upregulated and downregulated DEGs in *Cx. pipiens pallens* at GO level_2 between the low-temperature treatment and control groups. **d** KEGG annotations of the top 20 DEGs. A is the control group, and B is the low-temperature treatment group

We identified 2417 DEGs between the low-temperature-treated and control groups (Fig. 4a and Additional file 1: Table S3), of which 1316 were upregulated and 1101 were downregulated (Additional file 1: Table S4, S5). For all DEGs, the gene expression patterns were similar among biological replicates but significantly different between the low-temperature-treated and control groups (Fig. 4b). The GO annotations of these genes included 23 biological processes, 20 cellular components and 21 molecular functions, with the highest enrichment, observed for the terms cellular process, metabolic process and single organism process in the biological process category; in cell and cellular component in the cellular component category; and binding and catalytic activity in the molecular function category (Fig. 4c). The 20 top enriched KEGG pathways are shown in Fig. 4d. Under low-temperature stress, the DEGs were enriched in oxidative phosphorylation, followed by carbon metabolism, the tricarboxylic acid cycle and amino acid biosynthesis.

### Differentially expressed genes associated with gut bacterial community composition

**Fig. 5:**
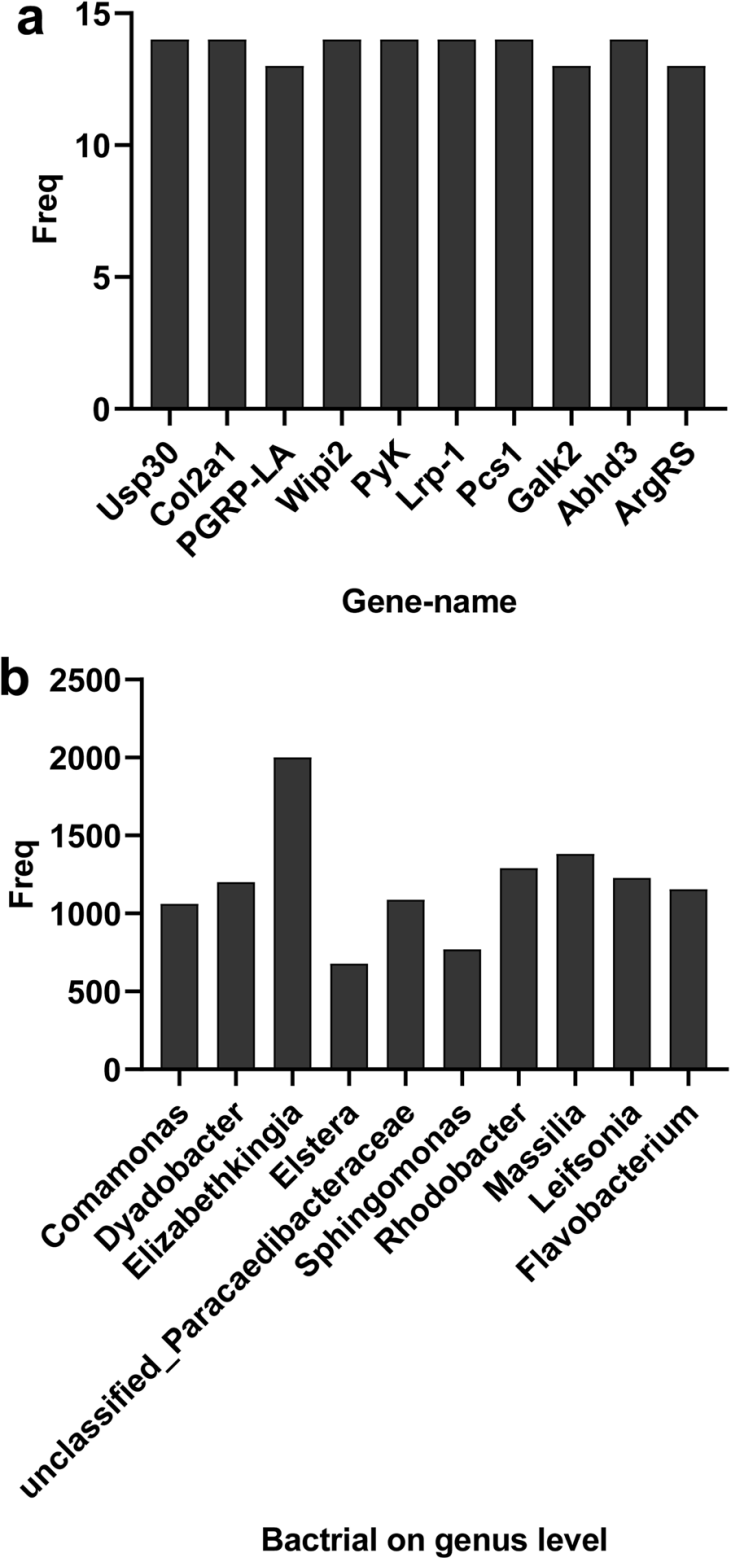
Relationship between DEGs and bacteria (genus level) in *Cx. pipiens pallens* treated at low temperature and the control group. **a** Top 10 DEGs associated with gut bacteria. **b** Top 10 bacterial genera associated with DEGs

Pearson correlation analysis was used to infer the relationship between genus-level microbial communities and host gene expression. A total of 87,012 pairs were explored, including 2417 genes and 36 genera, of which 16,297 pairs showed significant association (Additional file 1: Table S6). Figure 5a lists the top 10 candidate genes with genus-level relationships to bacterial communities. These genes have a wide range of functions, such as peptido glycan-recognition protein LA (PGRP-LA), which is mainly involved in immune responses; collagen alpha-1(II) chain (Col2a1), phospholipase ABHD3 (Abhd3), WD repeat domain phosphoinositide-interacting protein 2 (Wipi2), pyruvate kinase (PyK) and low-density lipoprotein receptor-related protein (Lrp-1) related to protein and lipid synthesis and metabolism, and probable citrate synthase 1 (Pcs1), which is mainly involved in energy metabolism. The top 10 genera identified by gene frequency pairing were: *Comamonas*, *Dyadobacter*, *Elizabethkingia*, *Elstera*, *unclassified_Paracaedibacteraceae*, *Sphingomonas*, *Rhodobacter*, *Massilia*, *Leifsonia* and *Flavobacterium* (Fig. 5b).

## Discussion

*Culex pipiens pallens* enters the diapause state in low-temperature environments to resist cold stress and maintain normal breeding of the race [4]. The density of overwintering mosquitoes determines the number of mosquitoes in the coming year and has an important impact on mosquito-borne diseases. In this study, the gut microbes of *Cx. pipiens pallens* were removed to the greatest extent possible by oral feeding with antibiotics, and this treatment did not affect the survival of adult mosquitoes [37]. Subsequently, we found that 7 °C was the lowest temperature at which the median survival time of antibiotic-treated *Cx. pipiens pallens* was the most significantly reduced, suggesting that the change in the lifespan of *Cx. pipiens pallens* at 7 °C might be related to the gut bacterial community.

By high-throughput sequencing of full-length 16S rRNA genes, we found significant differences in gut bacterial community structure between the control and treatment groups. Under low-temperature stress, the Shannon and Simpson indices of the gut bacterial community of *Cx. pipiens pallens* decreased, and the OTU abundance at the genus and species levels decreased, indicating a decrease in bacterial diversity. Low temperature decreased the abundance of *Elizabethkingia* and *Dyadobacter* and increased the abundance of *Aeromonas*, suggesting that low-temperature stress not only affected the species of gut bacteria but also affected the abundance of gut bacteria. *Elizabethkingia* showed a significant difference between the two groups, suggesting that bacteria of this genus may be sensitive to low-temperature environments. *Pseudomonas* can survive and thrive in the midgut of mosquitoes during several molting or ecdysis events during larval stages as well as hydrolytic processes during metamorphosis and finally transfer to adults [38]. In addition, *Pseudomonas* provides the host with amino acids, cofactors, vitamins and other essential nutrients [39]. Raza et al. [13] found that intestinal symbiotic bacteria promoted host resistance to low-temperature stress by stimulating the arginine and proline metabolic pathways of adult *Bactrocera dorsalis*. Therefore, based on our experimental results, we speculated that gut bacteria also played a role in the resistance of *Cx. pipiens pallens* to low-temperature stress. For example, gut bacteria may assist *Cx. pipiens pallens* in the digestion, absorption and utilization of sugar water to obtain energy for resistance to low-temperature stress [18, 40]. In addition, gut bacteria may increase the expression levels of some genes related to resistance to low-temperature stress in *Cx. pipiens pallens*, enabling these mosquitoes to resist low-temperature stress [13].

Transcriptome analysis showed that most of the DEGs were enriched in biological process groups during GO function analysis. Among them, cellular processes and metabolic processes accounted for a high proportion, consistent with the changes observed in *Aldrichina grahami* at low temperature [41]. In addition, biological processes such as response to stimuli, biological regulation and immune system processes also accounted for a large proportion at low temperature, indicating that the innate immune response of insects is related to low-temperature environments, and extreme temperature activates the innate immune response of insects [42, 43]. Binding and catalytic activities account for a majority of molecular functional groups, indicating that these pathways play a key role in temperature regulation [44]. Oxidative phosphorylation, the tricarboxylic acid cycle and glycolysis/gluconeogenesis were all associated with glucose metabolism among the KEGG pathways with relatively high enrichment, suggesting that *Cx. pipiens pallens* increased energy consumption to cope with low-temperature stress. Under low-temperature stress, the oxidative phosphorylation pathway is the most important metabolic pathway affecting *Cx. pipiens pallens*. The oxidative phosphorylation pathway is also an important part of the cold adaptation mechanism of *Liriomyza trifolii* pupae and the ghost moth *Hepialus xiaojinensis* [44, 45]. Cryoprotectants are an important substance used by insects to resist low-temperature stress, and carbon metabolism is closely related to the synthesis of carbohydrate cryoprotectants [42].

Studies have shown that some insects respond to low-temperature environments by promoting the expression of genes encoding cold resistance substances such as trehalose, HSPs, antioxidant enzymes and enzymes related to the synthesis and degradation of cryoprotectants [46–48]. HSPs are molecular chaperones produced by organisms in response to an environmental pressure that help correct the folding of amino acid chains and participate in the transmembrane transport of proteins [49]. One HSP70 and three HSP20 genes were significantly upregulated in the cold-adapted transcriptomes of *Cx. pipiens pallens* at 7 °C, suggesting that HSP70 and HSP20 were sensitive to low temperature. Some studies have found that cytochrome P450 is involved in the low-temperature response of many insects [50, 51]. In this study, 14 genes related to the cytochrome P450 pathway were upregulated in the transcriptome of *Cx. pipiens pallens* under low-temperature stress, suggesting that *Cx. pipiens pallens* may respond to low-temperature stress by triggering the cytochrome P450-mediated thermoregulation mechanism. Superoxide dismutase (SOD) is a metal enzyme that catalyzes the decomposition of superoxide anion radicals to hydrogen peroxide and oxygen in organisms [52]. SOD and other antioxidant enzymes play an important role in the overwintering process of *Cx. pipiens pallens* [53], and SOD-related genes were also identified in this study.

Finally, we inferred the relationship between gene-level microbial communities and host gene expression. Because our purpose was to explore the relationship between a certain bacteria or a certain type of bacteria and the gene expression level and the sequencing platform PacBio we adopted has a species annotation rate of 95% at the genus level and 60% at the species level, we propose that the genus level sequence information is more complete and more likely to be annotated with more accurate species information. Candidate genes related to bacterial communities may play an important role in the resistance of *Cx. pipiens pallens* to low-temperature stress. In *Drosophila* (Ddiptera), upregulated expression of several immune system genes was also detected at low temperature [42], and PGRP-LA is mainly involved in immune function, suggesting that insect immunity is related to low temperature. AFPs protect organisms from freezing by reducing freezing temperature and delaying the growth of ice, and they are highly abundant in some insects that avoid freezing in winter [46]. Lipids are an important component of insect overwintering energy [54]. Col2a1, Abhd3, Wipi2, PyK, Lrp-1 and other genes are mainly involved in protein and lipid synthesis and metabolism and may provide necessary lipids and proteins for *Cx. pipiens pallens* to resist low-temperature stress. Pcs1 may participate in the citric acid cycle and provide energy for *Cx. pipiens pallens* to resist low-temperature stress.

It is generally believed that the gut bacterial community interacts with the expression of certain genes in the intestinal tissue of the host to affect the host [55]. However, gut bacteria also influence gene expression in other tissues of the host. For example, colonization by the symbiotic fungus *Snodgrassella alvi* can activate the systemic immunity of honeybees and lead to upregulated expression of antimicrobial peptide genes in body fat [56]. As probiotics, *Erythrococcus* species can induce a protective immune response in *Oncorhynchus mykiss* (Walbaum) to effectively prevent vibriosis in rainbow trout [57], and *Erythrococcus* was also detected in this study. Our results also showed that there was a significant correlation between the gut bacterial community and DEGs, so we speculated that the response of *Cx. pipiens pallens* to low-temperature stress might be the result of combined effects of the gut bacterial community and gene expression.

## Conclusions

In this study, we found that low-temperature stress affected the abundance and diversity of the gut bacteria of *Cx. pipiens pallens*. At the same time, transcriptome data analysis under low-temperature stress revealed that the expression of genes related to biological processes such as metabolic processes, cellular processes, biological regulation, responses to stimuli and immune system processes was significantly upregulated, and the expression of some abiotic stress response genes suggested that *Cx. pipiens pallens* may respond to low-temperature stress via molecular chaperone activity, antioxidant defense, thermoregulation and other mechanisms. The results of Pearson’s correlation analysis showed a significant correlation between the gut micro-community at the genus level and several DEGs. Therefore, we speculated that the mechanism of adaptation of *Cx. pipiens pallens* to low-temperature stress may be the result of the interaction between the gut bacterial community and transcriptome, providing new insights for further study on the mechanism underlying the response of this species to low-temperature stress.

## Supplementary Information

**Additional file 1: Table S1.** Basic information of 16S rRNA sequencing of gut bacteria. **Table S2**. Basic information of transcriptome sequencing. **Table S3.** DEGs. **Table S4.** GO level_2 statistics. **Table S5.** Top 20 enriched KEGG pathways. **Table S6.** Pearson’s correlation analysis of the data and statistics.

## Data Availability

The datasets supporting the findings of this article are included within the paper and its Additional files. All of the transcriptome and 16S rRNA amplicon sequencing data have been deposited in NCBI (https://www.ncbi.nlm.nih.gov/sra) under the accession numbers PRJNA850617 and PRJNA850502.
