# Supplementary material for: Interactions between the gut micro-community and transcriptome of Culex pipiens pallens under low-temperature stress
Source: Parasit Vectors. 2023 Jan 12;16:12. doi: 10.1186/s13071-022-05643-7 (PMC9837946; doi:10.1186/s13071-022-05643-7)
Supplement: Supplementary file 1 — Additional file 1: Table S1. Basic information of 16S rRNA sequencing of gut bacteria. Table S2. Basic information of transcriptome sequencing. Table S3. DEGs. Table S4. GO level_2 statistics. Table S5. Top 20 enriched KEGG pathways. Table S6. Pearson’s correlation analysis of the data and statistics. [file 13071_2022_5643_MOESM1_ESM.zip › Additional file 1 Table S2.docx]

**Table S2** Basic information of transcriptome sequencing data of *Culex pipiens pallens* in control group and low-temperature treatment group

| Samples | Clean reads | Clean bases | GC (%) | Q30 (%) |
| --- | --- | --- | --- | --- |
| A1 | 48,661,544 | 7,174,645,018 | 52.69% | 92.32% |
| A2 | 48,588,832 | 7,164,422,376 | 53.17% | 92.61% |
| A3 | 44,210,356 | 6,519,300,555 | 52.75% | 92.61% |
| A4 | 48,516,794 | 7,155,633,781 | 53.14% | 92.59% |
| B1 | 47,366,254 | 6,985,387,528 | 50.66% | 92.39% |
| B2 | 50,100,412 | 7,391,877,750 | 50.86% | 92.29% |
| B3 | 48,736,600 | 7,195,663,758 | 51.54% | 92.20% |
| B4 | 44,189,310 | 6,526,022,776 | 48.35% | 92.69% |
| Total | 380,370,102 | 56,112,953,542 |  |  |

Note: GC (%) represents the total number of G and C in Clean base as a percentage of the total number of bases. Q30 (%) represents the percentage of bases with Qphred value greater than 30 in the total Raw base. The letter A represents the control sample, and the letter B represents the low-temperature treated sample.
