# Supplementary material for: Interactions between the gut micro-community and transcriptome of Culex pipiens pallens under low-temperature stress
Source: Parasit Vectors. 2023 Jan 12;16:12. doi: 10.1186/s13071-022-05643-7 (PMC9837946; doi:10.1186/s13071-022-05643-7)
Supplement: Supplementary file 1 — Additional file 1: Table S1. Basic information of 16S rRNA sequencing of gut bacteria. Table S2. Basic information of transcriptome sequencing. Table S3. DEGs. Table S4. GO level_2 statistics. Table S5. Top 20 enriched KEGG pathways. Table S6. Pearson’s correlation analysis of the data and statistics. [file 13071_2022_5643_MOESM1_ESM.zip › Additional file 1 Table S1.docx]

**Table S1** Microbial community composition of *Culex pipiens pallens* samples treated with control and low temperature

| Sample ID | Raw CCS | Clean CCS | Effective CCS | AvLen(bp) | Phylum | Class | Order | Family | Genus | Species |
| --- | --- | --- | --- | --- | --- | --- | --- | --- | --- | --- |
| C1 | 12,382 | 12,380 | 12,318 | 1,436 | 4 | 5 | 13 | 15 | 16 | 17 |
| C2 | 13,052 | 13,048 | 12,893 | 1,441 | 3 | 4 | 13 | 17 | 20 | 23 |
| C3 | 12,980 | 12,975 | 12,772 | 1,453 | 4 | 5 | 14 | 17 | 20 | 23 |
| C4 | 12,926 | 12,923 | 12,801 | 1,448 | 4 | 5 | 15 | 21 | 22 | 25 |
| T1 | 13,017 | 13,011 | 12,939 | 1,462 | 3 | 4 | 8 | 10 | 11 | 11 |
| T2 | 13,013 | 13,008 | 13,002 | 1,435 | 4 | 5 | 12 | 19 | 22 | 24 |
| T3 | 13,044 | 13,039 | 13,010 | 1,443 | 4 | 5 | 10 | 13 | 18 | 21 |
| T4 | 12,971 | 12,967 | 12,948 | 1,434 | 4 | 5 | 9 | 10 | 12 | 13 |
| Total | 103,385 | 103,351 | 102,683 |  | 4 | 5 | 18 | 28 | 36 | 41 |

Note: The letter C represents the control sample, and the letter T represents the low-temperature treated sample.
